# *Ganoderma lucidum* polysaccharides ameliorate lipopolysaccharide-induced acute pneumonia via inhibiting NRP1-mediated inflammation

**DOI:** 10.1080/13880209.2022.2142615

**Published:** 2022-11-14

**Authors:** Xuelian Zhang, Daoshun Wu, Yu Tian, Xiangdong Chen, Jin Lan, Fei Wei, Ye Li, Yun Luo, Xiaobo Sun

**Affiliations:** a Institute of Medicinal Plant Development, Peking Union Medical College and Chinese Academy of Medical Sciences, Beijing, China; b Beijing Key Laboratory of Innovative Drug Discovery of Traditional Chinese Medicine (Natural Medicine) and Translational Medicine, Institute of Medicinal Plant Development, Peking Union Medical College and Chinese Academy of Medical Sciences, Beijing, China; c Key Laboratory of Bioactive Substances and Resources Utilization of Chinese Herbal Medicine, Ministry of Education, Beijing, China; d NMPA Key Laboratory for Research and Evaluation of Pharmacovigilance, Institute of Medicinal Plant Development, Peking Union Medical College and Chinese Academy of Medical Sciences, Beijing, China; e Ganoherb (Fujian) Technology Corporation, Nanping, China

**Keywords:** Ganodermataceae, COVID-19, pneumonocyte apoptosis, anti-inflammatory

## Abstract

**Context:**

*Ganoderma lucidum* polysaccharides (GLP), from *Ganoderma lucidum* (Leyss. ex Fr.) Karst. (Ganodermataceae), are reported to have anti-inflammatory effects, including anti-neuroinflammation and anti-colitis. Nevertheless, the role of GLP in acute pneumonia is unknown.

**Objective:**

To explore the protective role of GLP against LPS-induced acute pneumonia and investigate possible mechanisms.

**Materials and methods:**

GLP were extracted and used for high-performance liquid chromatography (HPLC) analysis after acid hydrolysis and PMP derivatization. Sixty C57BL/6N male mice were randomly divided into six groups: Sham, Model, LPS + GLP (25, 50 and 100 mg/kg/d administered intragastrically for two weeks) and LPS + dexamethasone (6 mg/kg/d injected intraperitoneally for one week). Acute pneumonia mouse models were established by intratracheal injection of LPS. Haematoxylin and eosin (H&E) staining was examined to evaluate lung lesions. ELISA and quantitative real-time PCR were employed to assess inflammatory factors expression. Western blots were carried out to measure Neuropilin-1 expression and proteins related to apoptosis and autophagy.

**Results:**

GLP suppressed inflammatory cell infiltration. In BALF, cell counts were 1.1 × 10^6^ (model) and 7.1 × 10^5^ (100 mg/kg). Release of GM-CSF and IL-6 was reduced with GLP (25, 50 and 100 mg/kg) treatment. The expression of genes IL-1β, IL-6, TNF-α and Saa3 was reduced. GLP treatment also suppressed the activation of Neuropilin-1 (NRP1), upregulated the levels of Bcl2/Bax and LC3 and led to downregulation of the ratio C-Caspase 3/Caspase 3 and P62 expression.

**Discussion and conclusions:**

GLP could protect against LPS-induced acute pneumonia through multiple mechanisms: blocking the infiltration of inflammatory cells, inhibiting cytokine secretion, suppressing NRP1 activation and regulating pneumonocyte apoptosis and autophagy.

## Introduction

Coronavirus disease 2019 (COVID-19) does notable harm to mankind because of its high infectiousness, variability and lethality (Challen et al. 2021). Pneumonia and cytokine storms are the two most important pathological characteristics, especially in critically ill patients (Leisman et al. 2020). Moreover, it has been demonstrated that dexamethasone, a glucocorticoid anti-inflammatory agent, was the first drug to save lives during COVID-19 (Ledford 2020; Horby et al. 2021). Therefore, we assumed that drugs with anti-inflammatory efficacy may have potential therapeutic effects against COVID-19.

Acute pneumonia is a multiple respiratory disease characterised by diffuse inflammatory injury of the lungs, caused by various factors, such as lung tissue contusion, bacterial infections and viral infections. Activated immunocytes and excessive release of pro-inflammatory mediators are the essential features in the pathogenesis of acute pneumonia (Bakowitz et al. 2012). In addition, endotoxins stimulation can activate pro-inflammatory mediators release and result in acute pneumonia. As one of the bioactive components of the cell wall of Gram-negative (G-) bacteria, lipopolysaccharide (LPS) is a potent endotoxin widely used in *in vivo* or *in vitro* models of acute pneumonia (Ko et al. 2021; Abd El-Fattah et al. 2022). In the animal models stimulated by LPS, the typical characteristics of acute pneumonia patients are detected. Amounts of leukocytes in mice BALF increased dramatically (Lee et al. 2020) and various inflammatory factors, such as interleukin (IL)-1β, IL-6 and tumour necrosis factor (TNF)-α are secreted increasingly (Zhu et al. 2018; Wu et al. 2022). In addition, LPS treatment can induce cell apoptosis and autophagy (Du et al. 2020; Chen et al. 2021; Yang et al. 2021).

Neuropilin-1 (NRP1), an encoded transmembrane protein, is expressed in many human tissues, notably in the lung (Saiz et al. 2021). In addition, NRP1 is a surface receptor found on cancer cells, immune cells and many other cell types (Chuckran et al. 2020; Liu et al. 2020). Mainly, it has been reported that NRP1 was found to be effective in diverse immune cells, including macrophages, dendritic cells and T cell subpopulations, which indicated the vital effect of NRP1 on the regulation of immune response as well as respiratory diseases (Tordjman et al. 2002; Roy et al. 2017; Wilson Ariel et al. 2018). Notably, it has been reported that SARS-CoV-2 Spike protein can bind to the b1b2 domain of NRP1, and NRP1 is an essential mediator to assist the entry of SARS-CoV-2 into normal cells and lead to cell infectivity (Cantuti-Castelvetri et al. 2020; Daly James et al. 2020). At present, the inhibition of NRP1 expression is considered an effective strategy for blocking viral infectivity and spreading (Kolarič et al. 2022).

*Ganoderma lucidum* (Leyss. ex Fr.) Karst. is a well-known medicinal fungus and globally used as a dietary supplement owing to its immunoregulatory and antitumor potential (Lin and Zhang 2004). As one of the most important constituents of *Ganoderma lucidum*, *Ganoderma lucidum* polysaccharides (GLP) exert diverse biological activities, including antitumor, immunomodulatory, antiviral and antidiabetic activities (Lu et al. 2020). Moreover, GLP can prevent bleomycin-induced pulmonary fibrosis (Chen et al. 2016) and suppress lung cancer (Hsu et al. 2020; Qiu et al. 2021). However, so far, no research regarding the effect of GLP on acute pneumonia has been reported. To explore the preventive action of GLP on acute pneumonia, we established the murine LPS-induced acute pneumonia model to determine the anti-inflammatory effect and explore its potential mechanism.

## Materials and methods

### Chemicals and reagents

*Ganoderma lucidum* spore powder was obtained from Ganoherb (Fujian) Technology Corporation (Fujian, China). The chemical standards of D-glucose, L-fucose, D-galactosamine, D-galacturonic acid, D-ribose, D-xylose, D-mannose, D-galactose, L-rhamnose, D-glucuronic acid and D-arabinose were purchased from Shanghai Yuanye Biotechnology Co., Ltd. Analytical grade trifluoroacetic acid, alcohol and 1-phenyl-3-methyl-5-pyrazolone were obtained from Shanghai Macklin Biochemical Co., Ltd. High-performance liquid chromatography (HPLC)-grade methanol was purchased from Thermo Fisher Scientific Inc. LPS (*Eschericha coli* serotype O127:B8) was purchased from Sigma-Aldrich Inc. Dexamethasone sodium phosphate injection was obtained from Beijing Solarbio Science & Technology Co., Ltd. Antibodies of Caspase 3 (1:1000, ab184787), C-Caspase 3 (1:1000, ab214430), Bcl-2 (1:1000, ab182858), Bax (1:1000, ab32503), Neuropilin-1 (1:1000, ab81321), P62 (1:1000, ab109012), LC-3 (1:1000, ab192890) and GAPDH (1:1000, ab8245) were obtained from Abcam (Cambridge, UK). Secondary goat anti-mouse and anti-rat IgG were purchased from Cell Signaling Technology (Danvers, MA, USA). BCA protein assay kits were purchased from Beyotime Biotechnology (Shanghai, China). Enzyme linked immunosorbent assay (ELISA) kits of GM-CSF and IL-6 were purchased from ABclonal Biotechnology Co., Ltd. cDNA reverse-transcription kits were procured from Takara Biotechnology Co., Ltd. (Dalian, China).

### Preparation of standard solution and crude GLP extract

d-Glucose, l-fucose, d-galactosamine, d-galacturonic acid, d-ribose, d-xylose, d-mannose, d-galactose, l-rhamnose, l-glucuronic acid and d-arabinose were mixed in distilled water to prepare a standard solution with the concentration of 0.33 mg/mL. The solutions prepared were hold at 4 °C so as to analyse subsequently. A total of 20.0 g of unbroken *Ganoderma lucidum* spore powder was soaked in 150 mL of alcohol (95%, w/v) overnight, and the mixture was filtered to remove fat and other ethanol-soluble substances. In a round-bottom flask, 300 mL of water was added and the residue was extracted at 80 °C for 2 h to collect the filtrate while being hot. After three extractions under the same conditions, the filtrates were combined and concentrated under vacuum. The concentrate and 10 mL of water were mixed and then added to 80 mL of erthanol (95%, w/v) slowly while stirring. The ethanol mixture was incubated at 4 °C overnight. With the centrifugal speed of 5000 rpm lasting for 20 min, the mixture was separated into layers and the precipitate was retained and dried in an oven to obtain *Ganoderma lucidum* crude polysaccharides (Li et al. 2020; Wen et al. 2022).

### Acid hydrolysis of GLP

To a 1 mL volumetric flask was added 30.0 mg of prepared crude polysaccharides, following by the addition of distilled water so that the crude polysaccharide aqueous solution was obtained. The crude polysaccharide aqueous solutions (500 μL) were incubated with trifluoroacetic acid (500 μL, 0.4 mol/L) in a 5 mL stoppered test tube under nitrogen and the mixtures were allowed to hydrolyse at 110 °C for 70 min. After the hydrolysates were cooled down to room temperature, an equal volume of methanol was added and then the mixture was dried under nitrogen flow to remove trifluoroacetic acid. The drying process above was repeated for three times until the trifluoroacetic acid was completely removed. At last, the hydrolysis product was redissolved in distilled water to fix the volume to 1 mL for subsequent PMP derivatization (Xue et al. 2018; Wen et al. 2022).

### PMP derivatization of the mixed standards solution and GLP hydrolysate

The 1-phenyl-3-methyl-5-pyrazolone (PMP) derivatization method was as follows (Dai et al. 2010; Xue et al. 2018): The monosaccharide standards solution (100 μL) and sodium hydroxide solution (100 μL, 0.6 mol/L) were mixed and shaken well. Then 100 μL of the mixture was added into a 5 mL stoppered test tube, followed by the addition of PMP methanolic solution (100 μL, 0.5 mol/L). The above solution was allowed to cool to room temperature after heated at 70 °C for 100 min. Subsequently, hydrochloric acid solution (120 μL, 0.3 mol/L) was added, following by the addition of distilled water to dilute to 2 mL precisely. And then, an equal volume of chloroform was added, and the mixture was shaken vigorously. The chloroform layer was discarded and the extraction process above was repeated for three times to remove the excess PMP reagents thoroughly. Finally, the upper aqueous phase was filtered through a 0.22 μm membrane for HPLC analysis.

The PMP derivatization of GLP hydrolysate was consistent with the procedures above.

### Components analysis of GLP

HPLC was employed to analyse PMP derivatives obtained. A Waters 2690 HPLC system was employed with a Waters 2487 dual-wavelength detector and a Luna Omega Polar C18 100 Å column (5 μm, 250 × 4.6 mm) equipped. The mobile phase, consisting of (A) phosphate buffer (0.1 mol/L, pH 6.7) and (B) methanol, was used to separate the monosaccharides, and separation was achieved using isocratic elution. Temperature of the column was chosen as 30 °C, and isocratic elution (A vs. B = 61.5: 38.5) at a flow rate of 1 mL/min was used as the mobile phase for 60 min. The UV detection wavelength of 254 nm and 10 μL of the injection volume were chosen. Compared with those of the corresponding standards, the chromatographic data revealed the compositions of GLP.

### Ethical statement

All animal assays and experimental protocols were reviewed and approved by the Animal Ethics Committee of the Institute of Medicinal Plant Development (IMPLAD), Chinese Academy of Medical Sciences (April 2019, SYXK 2017-0020).

### Grouping and treatment of animals

A total of 60 C57BL/6N male mice (weight 18–22 g) were raised at the SPF Animal Centre of IMPLAD with adequate supply of food and water. In addition, the room temperature was kept at approximately 22 ± 1 °C and a 12 h light-dark cycle was set. The mice were randomly divided into six groups: (i) sham group (normal saline, i.g.), (ii) LPS model group (normal saline, i.g.), (iii) LPS + GLP (25 mg/kg/day, i.g.)-treated, (iv) LPS + GLP (50 mg/kg/day, i.g.)-treated, (v) LPS + GLP (100 mg/kg/day, i.g.)-treated and (vi) LPS + dexamethasone (6 mg/kg/day, i.p.)-treated. Mice in groups i–v were administered intragastrically for two weeks, whereas mice in group vi were injected intraperitoneally for one week. All mouse weights were measured every two days. The acute pneumonia models were established by intratracheal injection of LPS (10 mg/kg) in 25 μL saline. At the same time, mice in the sham group were injected with the same amount of normal saline into trachea. At 2 h before LPS inoculation, drugs were given for the last time. On the night before the last experiment, the mice were fasted. After 24 h of LPS treatment, samples of blood were obtained from the inner canthus. Serum was collected with the centrifugation speed of 3000 rpm and maintained at −80 °C for subsequent analysis.

### Measurements of inflammatory cytokines

With instructions of the manufacturer, levels of inflammatory cytokines in serum, including IL-6 and GM-CSF, were measured with ELISA kits (Abclonal).

### Acquisition and analysis of BALF

Pre-cooled PBS (1 mL) was injected into trachea to gather bronchoalveolar lavage fluid (BALF). The collected fluid was centrifuged for 10 min at 1500 rpm at 4 °C. Red blood cell lysis buffer was added after the supernatant was discarded. The suspended liquid was then centrifuged again. Total leukocyte counts were determined using a cell counter (Zhang H et al. 2020).

### Histological analysis of the lung tissues

The lung tissues of sacrificed animals were collected, washed in cold PBS and weighed. The calculation formula of lung index is as follows (Chen et al. 2016):

Lung index=(wet lung weight)/body weight×100%

After excision, the lung tissues were placed into 4% paraformaldehyde and immersed for 2 d. Then, the samples were treated according to the methods reported previously (Chen et al. 2016; Zhang et al. 2020) as the following steps: (1) dehydrated using ethanol and xylene; (2) embedded in paraffin; (3) cut into 5 μm sections, and (4) stained with haematoxylin and eosin (H&E). Images were captured using an automatic scanning microscope (Leica Aperio CS2).

### Quantitative real-time PCR

Total frozen tissues RNA from the lung was extracted using TRIzol reagent (TransGen Biotech) according to the manufacturer’s instructions. Total RNA (2 μg) was reverse-transcribed into cDNA using PrimeScript^TM^ RT Master Mix. Cycle-time values were obtained by performing real-time PCR (RT-PCR) with Power SYBR Green PCR Master Mix (Takara Bio Inc., Kusatsu, Japan). As in our previous study (Luo et al. 2020), the 2^−ΔΔCt^ method was adopted for determining relative expression levels in each group. Primers (Table 1) were designed using Premier Primer Software 6.0 (Premier Biosoft International, Palo Alto, CA, USA).

**Table 1.** Primers used for quantitative real-time PCR.

Gene	Primer sequence (5′ to 3′)
Rn18s	F: TTCGGAACTGAGGCCATGATT
	R: TTTCGCTCTGGTCCGTCTTG
IL-1β	F: GCAACTGTTCCTGAACTCAACT
	R: ATCTTTTGGGGTCCGTCAACT
TNF-α	F: CCCTCACACTCAGATCATCTTCT
	R: GCTACGACGTGGGCTACAG
IL-6	F: TAGTCCTTCCTACCCCAATTTCC
	R: TTGGTCCTTAGCCACTCCTTC
Saa3	F: TGCCATCATTCTTTGCATCTTGA
	R: CCGTGAACTTCTGAACAGCCT

### Western blot

In an ice bath, lung tissues were lysed with a tissue protein extraction kit (Pierce Chemical Co., Rockford, IL), USA) and a protease inhibitor (0.1 mM) was added. To determine the protein concentration, we used a BCA kit (Jiangsu Cowin Biotech Co., Ltd., Taizhou, China). With 10% sodium dodecyl sulphate polyacrylamide gel electrophoresis, protein samples were separated and transferred onto nitrocellulose membranes (Luo et al. 2021). Immunoblotting was carried out with enhanced chemiluminescence (ECL) kit. Calculation of the band intensities was conducted using Gel Pro software (Media Cybernetics, Rockville, MD, USA).

### Statistical analysis

Statistical analysis was performed using the GraphPad Prism 6.0. When comparing more than two groups, all data are expressed as mean ± SEM, followed by one-way ANOVA with Tukey’s multiple comparison *post hoc* test. When values of *p* < 0.05, differences were recognized statistically significant.

## Results

### Compositions of GLP

**Figure 1.**
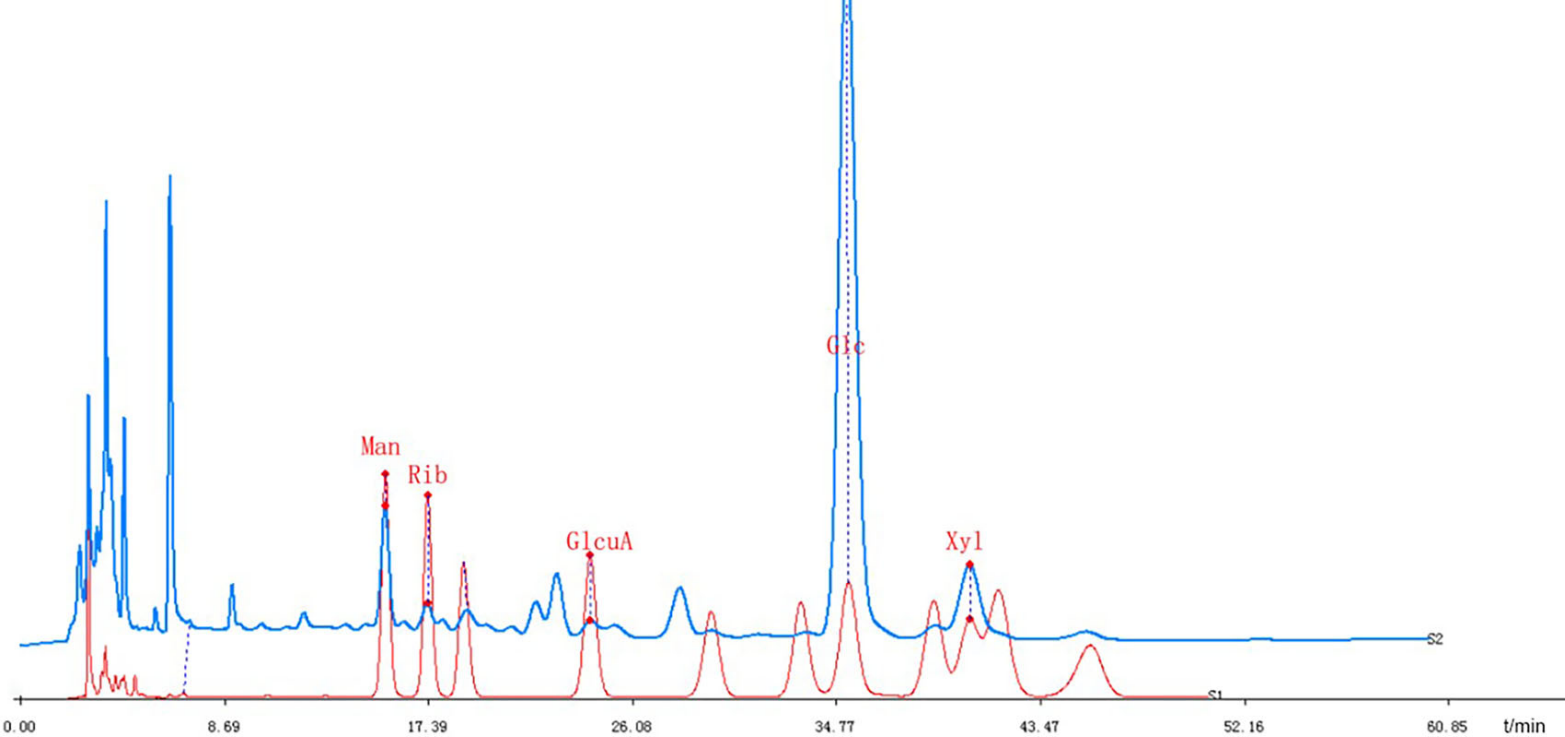
HPLC chromatogram of the monosaccharide composition of GLP at 254 nm.

HPLC analysis identified five monosaccharides from the GLP hydrolysate: glucose, mannose, ribose and glucuronic acid in the content ratio of 44:11:1:1, with relative contents of 2.64%, 0.247%, 0.061% and 0.058%, respectively, and a small amount of xylose. The GLP hydrolysate was identified by comparing its retention times with those obtained by injecting mixed standards under the same conditions (Figure 1).

### Effect of GLP on mice body weight and lung index

**Figure 2.**
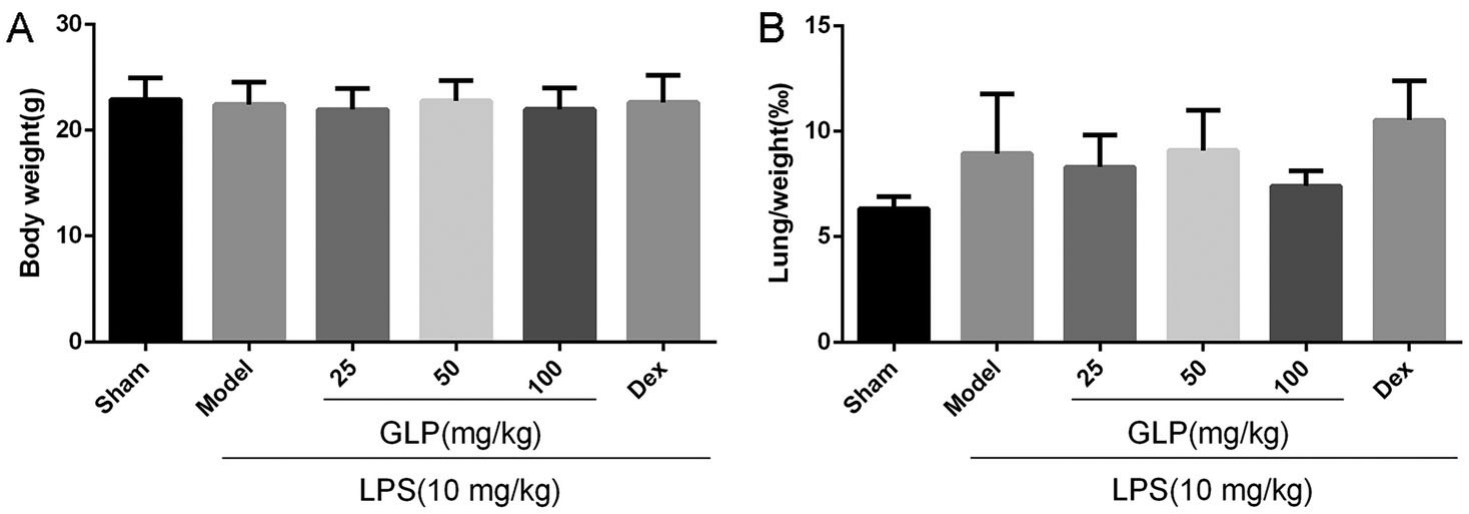
Body weight and lung index were collected and analysed. (A) Body weight of each group. (B) Lung index of each group (lung index = weight of wet lung/body weight × 100%). Data are present as mean ± SD (*n* = 10).

As displayed in Figure 2, no marked changes were found in body weight and lung index among the six groups, indicating that LPS exposure and administration of different doses of GLP had no effect on mice body weight as well as lung index.

### Effect of GLP on amounts of leukocytes in mice BALF

**Figure 3.**
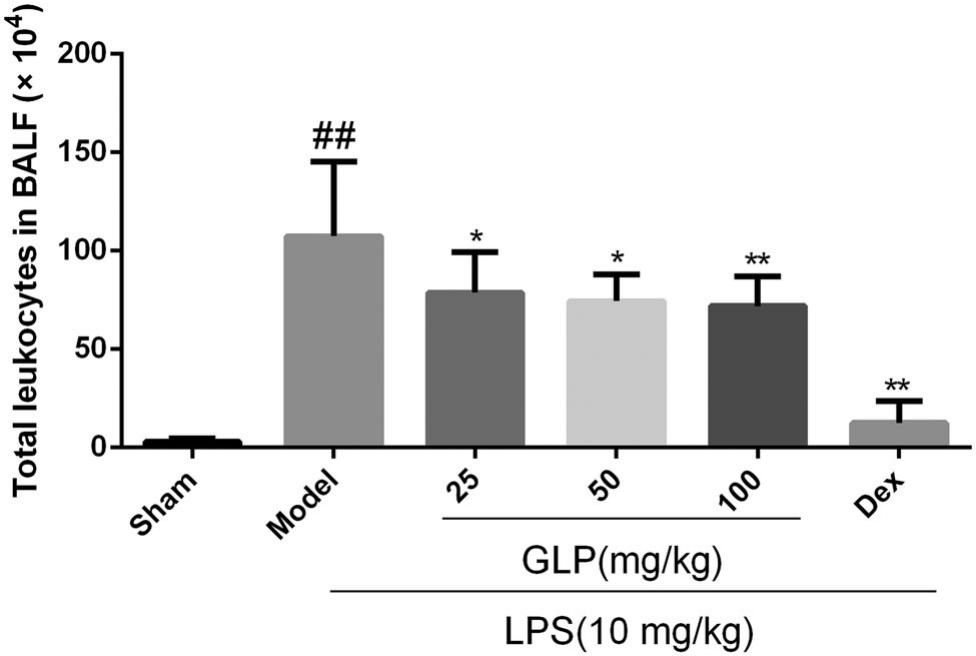
GLP suppressed the aggregation of leukocytes in BALF. Data are present as mean ± SD (*n* = 10). ^##^*p* < 0.01 as compared to Sham; *p < 0.05, **p < 0.01 as compared to Model.

To understand the role of GLP in LPS-induced acute pneumonia, we measured the leukocytes count in the BALF (Figure 3). In comparison with the sham, the total number of leukocytes (1.1 × 10^6^ cells) significantly increased in LPS-treated lungs (*p* < 0.01). However, BALF cell count (7.1 × 10^5^ cells) was markedly reduced after GLP treatment (100 mg/kg) compared with the model group (*p* < 0.01). Thus, GLP may alleviate LPS-induced lung inflammatory injury by inhibiting the elevation and aggregation of inflammatory cells.

### Effect of GLP on the morphological changes of mice with acute pneumonia

**Figure 4.**
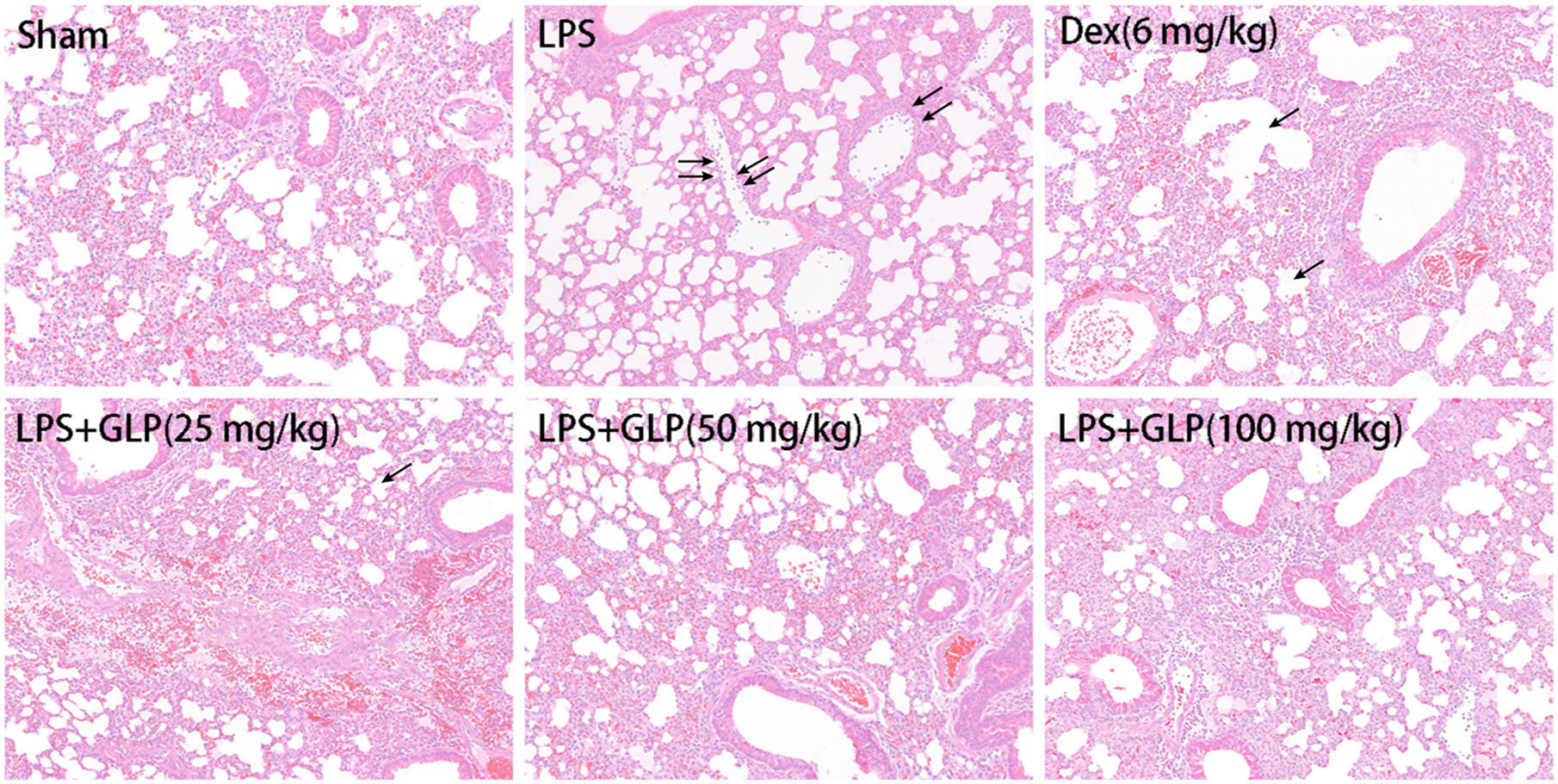
GLP alleviated LPS-induced histopathologic changes in lung tissues. After 24 h LPS treatment, lungs were cut off and stained with H&E for histopathology analysis (magnification 100×). Arrow: infiltration of inflammatory cells.

Inflammatory cell infiltration and pulmonary edoema are the main features of inflammatory response (Bakowitz et al. 2012). To assess the role of GLP in fighting against LPS-induced acute pneumonia, H&E staining was performed to detect histopathological changes in lung tissues following LPS or GLP treatment. As shown in Figure 4, in comparison to sham group, LPS induction increased the infiltration of inflammatory cells into the alveolar spaces and the occurrence of pulmonary edoema. On the contrary, the morphological changes were markedly suppressed after GLP treatment. The result showed that GLP had a protective role in preventing against LPS-induced acute pneumonia.

### Effect of GLP on the release of pro-inflammatory cytokines of mice with acute pneumonia

**Figure 5.**
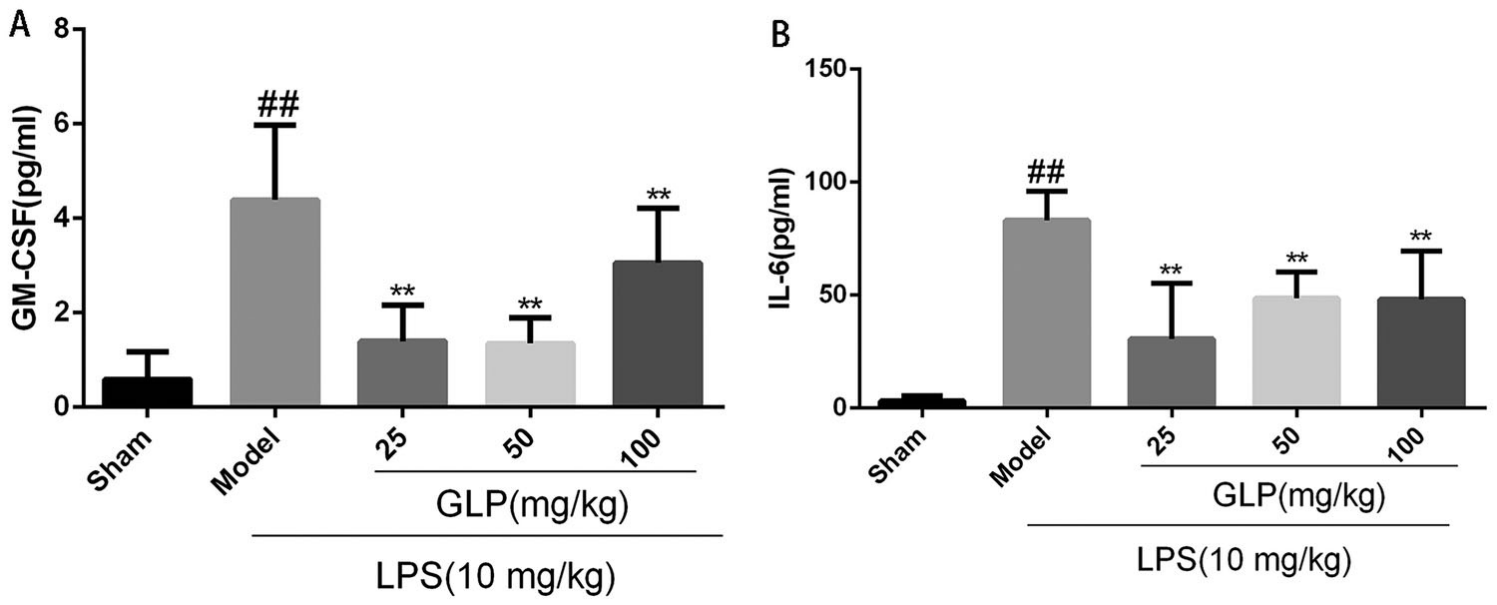
GLP downregulated the release of pro-inflammatory cytokines. (A) GM-CSF expression in plasma; (B) IL-6 expression in plasma. Data are present as mean ± SD (*n* = 10). ^##^*p* < 0.01 as compared to Sham; **p < 0.01 as compared to Model.

ELISA was used to determine the role of GLP in the regulation of inflammatory response (Figure 5). The expression of GM-CSF and IL-6 was markedly upregulated in mice suffering from acute pneumonia, suggesting that LPS could stimulate lung inflammation and injury (*p* < 0.01). However, in comparison to LPS-induced model group, the expression of GM-CSF and IL-6 in GLP-treated (25, 50 and 100 mg/kg) mice decreased significantly (*p* < 0.01). These results showed that GLP could reduce LPS-induced inflammatory response *via* modulating the release of plasma pro-inflammatory cytokines.

### Effect of GLP on the inflammation-related genes expression in mice with acute pneumonia

**Figure 6.**
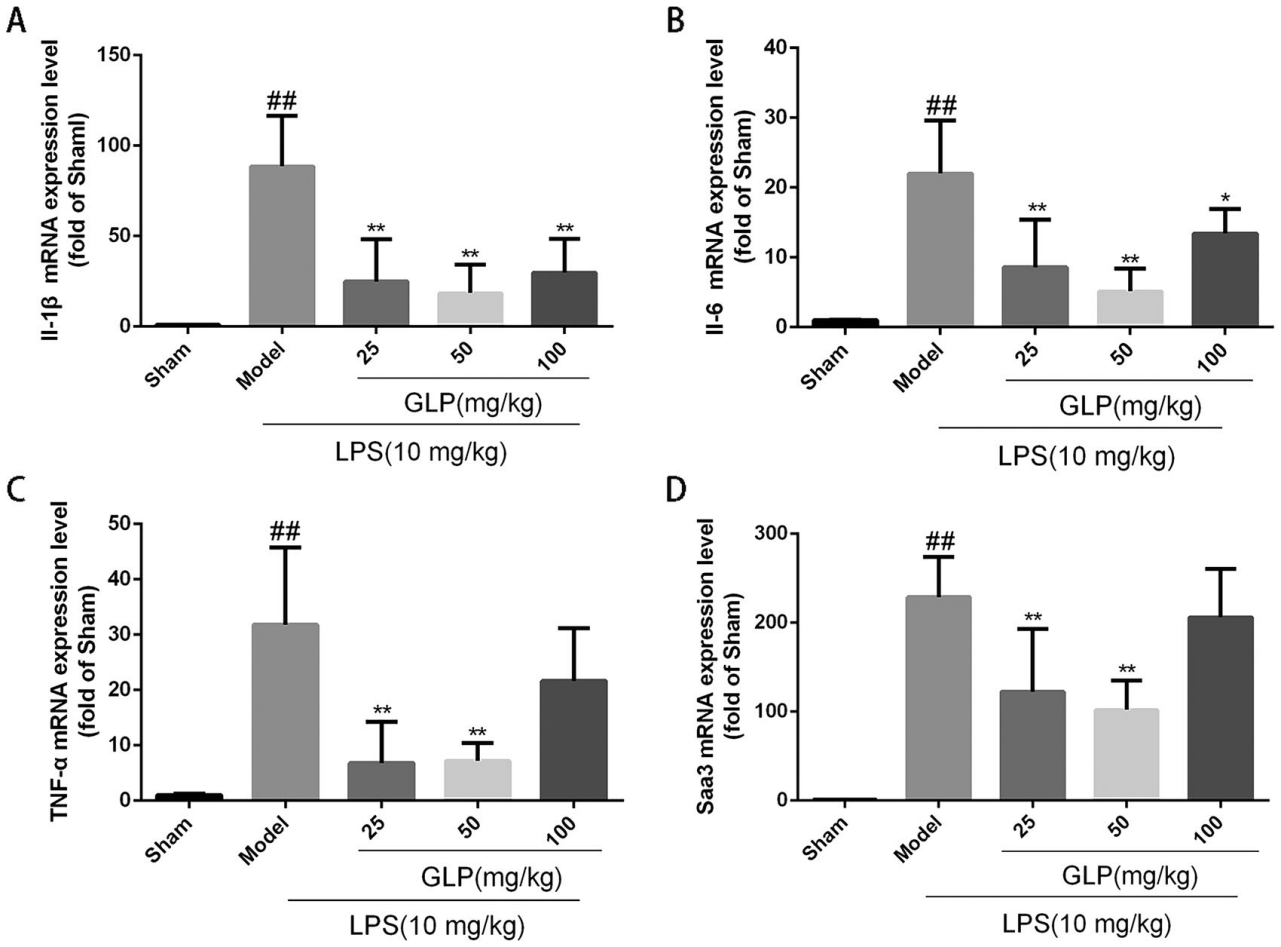
GLP attenuated the expression of pulmonary inflammation-related genes. (A) IL-1β mRNA expression level in serum; (B) IL-6 mRNA expression level in serum; (C) TNF-α mRNA expression level in serum; (D) Saa3 mRNA expression level in serum. Data are present as mean ± SD (*n* = 10). ^##^*p* < 0.01 as compared to Sham; *p < 0.05, **p < 0.01 as compared to Model.

To further clarify the underlying anti-inflammatory mechanisms of GLP, the relative mRNA expression of pulmonary IL-1β, IL-6, TNF-α as well as Saa3 was measured in the lung tissue by RT-PCR. As shown in Figure 6, in comparison to the sham group, the mRNA expression of pulmonary IL-1β, IL-6, TNF-α and Saa3 in the LPS-induced group was markedly increased (*p* < 0.01). Meanwhile, GLP (25, 50 mg/kg) significantly suppressed the expression levels of these genes (*p* < 0.01). These results suggested that GLP might efficiently reduce the activation of inflammation-related factors *via* the inhibition of the pulmonary inflammatory genes IL-1β, IL-6, TNF-α and Saa3, which were consistent with the changes of plasma inflammatory cytokines release.

### Effect of GLP on protein expression related to acute pneumonia

**Figure 7.**
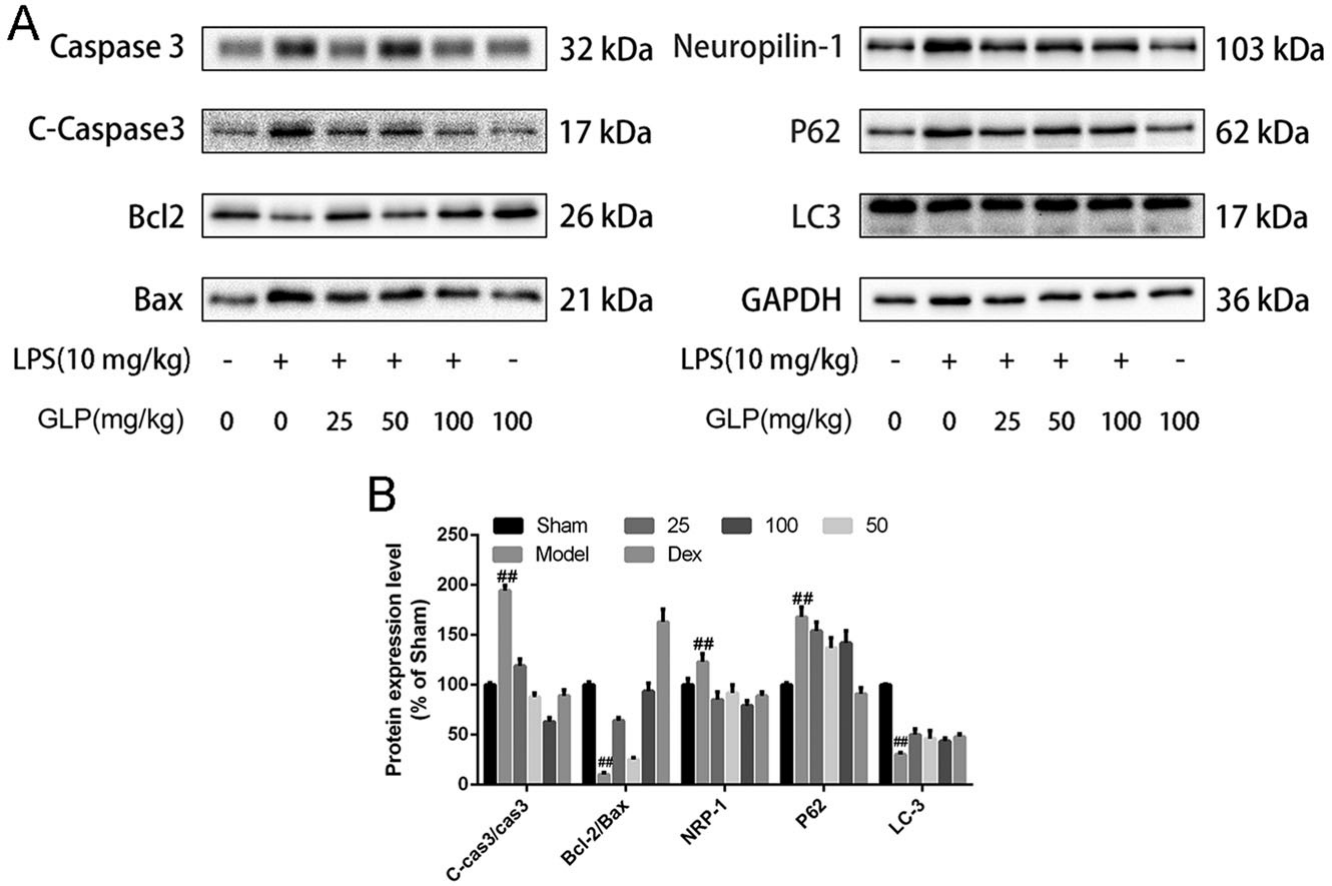
GLP regulated the expression of cell apoptosis and autophagy. (A) Western blot analysis of the representative proteins; (B) quantification of the expression of C-Caspase 3/Caspase 3, Bcl-2/Bax, NRP-1, P62, LC-3. GAPDH was used as a control. Data are present as mean ± SD (*n* = 10). ^##^*p* < 0.01 as compared to Sham; *p < 0.05, **p < 0.01 as compared to Model.

As apoptosis and autophagy are critical for the repair of damaged tissues (Song et al. 2017), we employed western blot to analyse the levels of proteins related to LPS-induced apoptosis and autophagy. First, we analysed the expression of Caspase 3, C-Caspase 3, Bcl-2 and Bax to detect cell apoptosis (Figure 7(A)). As represented in Figure 7(B), the ratio of C-Caspase 3/Caspase 3 and Bcl2/Bax remarkably increased and decreased in the LPS-treated group, respectively (*p* < 0.01). Nevertheless, pre-treatment with GLP at the dose of 25 and 50 mg/kg reversed these effects significantly (*p* < 0.01). To investigate the impact of GLP on autophagy in acute pneumonia mice, we evaluated the protein levels of LC3 and P62. Treatment with GLP led to a marked downregulation of the expression of P62 proteins, while that of LC3 was upregulated (*p* < 0.05). Moreover, the expression of NRP1, the cellular receptor of SARS-CoV-2, was increased significantly in the lung of LPS-induced group (*p* < 0.01), whereas treatment with GLP effectively reversed this effect (*p* < 0.05). Overall, these results suggested that GLP inhibited apoptosis, promoted autophagy and suppressed the expression of NRP1 to relieve LPS-induced acute pneumonia.

## Discussion

To date, hundreds of millions of infections and millions of deaths have been reported worldwide due to SARS-CoV-2 infection. COVID-19 is primarily a respiratory infectious disease characterized by acute pneumonia. As the leading cause of acute respiratory distress syndrome (ARDS), acute pneumonia has led to high morbidity and mortality in critically ill patients (Matthay and Zemans 2011). Currently, antibiotics are the most commonly prescribed therapeutic agents for treating patients with acute pneumonia. However, the abuse of antibiotics has led to antimicrobial resistance (MacGowan 2008), and therefore, the requirement of non-antibiotic drugs with marked anti-inflammatory action is urgent. *Ganoderma lucidum*, a traditional Chinese medicine, has been used for the treatment of multiple diseases in clinics (Lu et al. 2020; Ahmad et al. 2021). The compositions of *Ganoderma lucidum* are very complex, while the main biologically active components is GLP, which has been found to possess many therapeutic effects, including anticancer (Sohretoglu and Huang 2018), immunoregulatory (Seweryn et al. 2021), antioxidant (Zhao et al. 2012), antidiabetic (Chen et al. 2020; Shaher et al. 2020), antibacterial (Zhang et al. 2019) and neuroprotection (Cai et al. 2017) activities. In addition, earlier studies have suggested that GLP could prevent bleomycin-induced pulmonary fibrosis (Chen et al. 2016) and suppress lung cancer (Qiu et al. 2021). However, not many studies have looked at the anti-inflammatory properties of GLP in acute pneumonia. LPS has been used to induce an inflammatory response and establish an acute pneumonia model *in vivo* or *in vitro* (Zhang et al. 2020; Zhong et al. 2022). For this research, mouse models of acute pneumonia were built *via* the intratracheal injection of LPS and we explored the beneficial effects and underlying mechanisms of GLP in the treatment of LPS-induced acute pneumonia.

GLP were prepared by ethanol extraction for subsequent pharmacodynamic studies, and the constituents were analysed by HPLC. Studies have shown that inflammatory cell infiltration was a critical characteristic of acute pneumonia and an increasing number of inflammatory cells in BALF was observed in LPS-induced acute pneumonia (Zhang et al. 2020; Zhong et al. 2022). GLP attenuated the increase in leukocytes in the BALF and protected against LPS-induced acute pneumonia in the present study. Hence, GLP have excellent potential in treating LPS-challenged mice with acute pneumonia. Pulmonary edoema is a typical morphological change in acute pneumonia, and the lung index and body weight are used as indices of pulmonary edoema (Bakowitz et al. 2012; Song et al. 2021). Body weight and lung index were recorded and calculated, and no significant differences were found among all the groups, but the results of H&E staining revealed lung damage and pulmonary edoema in LPS-challenged mice. Therefore, GLP markedly attenuated LPS-induced pathological lung injury. To further understand the role of GLP in anti-inflammation, the expression of pro-inflammatory and inflammatory cytokines was examined.

After LPS was injected to induce acute pneumonia, a robust inflammatory response with cytokine storms, such as increases in GM-CSF, TNF-α, Saa3 and interleukins, was found (Lou et al. 2019; Zhang et al. 2020; Song et al. 2021). GLP protected against acute pneumonia through anti-inflammatory effect. ELISA results indicated that GLP significantly attenuated the LPS-induced increase in GM-CSF and IL-6 levels. RT-PCR further identified that GLP significantly inhibited the expression levels of IL-1β, IL-6, TNF-α and Saa3. These results are in accordance with previous reports and suggest that GLP inhibited the generation of inflammatory cytokines stimulated by LPS, which showed GLP had a protective effect on the prophylactic treatment of the inflammatory response.

Inflammation is closely correlated with apoptosis and can result in the induction of cell apoptosis (Li S et al. 2020). Autophagy can inhibit or induce apoptosis to regulate cell survival or death (Song et al. 2017). Apoptosis and autophagy play essential roles in the pulmonary inflammatory response caused by infection and irritation (Li et al. 2020). Previous studies have reported that GLP could downregulate the levels of pro-apoptotic proteins Caspase 3, C-Caspase 3 and Bax and increase the anti-apoptotic protein level of Bcl-2 in STZ-induced diabetic rats and mice with pneumonia (Yang et al. 2010; Song et al. 2019). The present study demonstrated that GLP exhibits anti-inflammatory effect by suppressing the ratio of C-Caspase 3/Caspase 3 and increasing the Bcl-2/Bax ratio. Notably, inhibition of autophagy is one of the primary mechanisms that result in pneumonia (Gassen et al. 2021). Our findings indicated that the level of P62 was downregulated, and the level of LC3 was elevated after GLP administration, suggesting the promotion of cell autophagy. SARS-CoV-2 enters cells with recognition and binding to Spike (S) protein receptors, and the angiotensin-converting enzyme 2 (ACE2) receptor is the main protein controlling viral entry (Shang et al. 2020). However, another mediator, Neuropilin 1 (NRP1), was also confirmed to be the channel protein contributing to the entry of the virus (Cantuti-Castelvetri et al. 2020; Daly James et al. 2020). The NRP1 protein is consisted with a small intracellular cytoplasmic domain, followed by a transmembrane region and a long N-terminal extracellular domain (Chaudhary et al. 2014). NRP1 is diffusely expressed across a variety of cells and tissues within the central nervous and vasculature systems. Notably, NRP1 is expressed more restrictedly in the immune system. In this study, NRP1 expression was reduced on GLP treatment. In short, GLP could downregulate NRP1 expression, promote cell autophagy and inhibit cell apoptosis to protect against acute pneumonia. Hence, we hypothesized that GLP blocked viral entry and reduced the inflammatory response to inhibit the spread of COVID-19.

Altogether, this study indicated that GLP could protect against LPS-induced acute pneumonia *via* diverse mechanisms, including the inhibition of inflammatory cell infiltration, reduction of cytokine secretion, downregulation of NRP1 expression and suppression of pneumonocyte apoptosis and autophagy. Based on these results, we suggest that GLP are a potential agent for treating SARS-CoV-2. However, with the data in hand, we are not sure whether GLP directly or indirectly regulated the downregulation of NRP1. In future studies, we will investigate the effects of different components of GLP and identify the major active ingredients. Moreover, further study is needed to explore the molecular mechanism of GLP in regulating NRP1.

## Conclusions

Collectively, we conducted multiple experiments to explore the protective effect and the underlying mechanisms of GLP on LPS-induced acute pneumonia. These results suggest that GLP has the advantage of preventing the potential damage from LPS-induced acute pneumonia. Pre-treatment with GLP significantly attenuated acute pneumonia by inhibiting inflammatory cell infiltration, reducing cytokine secretion, downregulating NRP1 expression and suppressing pneumonocyte apoptosis and autophagy. Therefore, GLP are suggested to be a potential anti-inflammatory agent and have efficacy in preventing acute pneumonia.
